# Does sexual Intimate Partner Violence (IPV) increase risk of multiple high-risk fertility behaviours in India: evidence from National Family Health Survey 2015–16

**DOI:** 10.1186/s12889-022-14289-0

**Published:** 2022-11-15

**Authors:** Milan Das, Csaba G. Tóth, Neha Shri, Mayank Singh, Babul Hossain

**Affiliations:** 1 International Institute for Population Sciences, Mumbai, India; 2 Research Fellow, Centre for Economic and Regional Studies, Budapest, Hungary; Corvinus Institute for Advanced Studies, Budapest, Hungary

**Keywords:** Sexual Intimate Partner Violence, Multiple high-risk fertility behaviour, NFHS, India

## Abstract

**Background:**

One in three women from lower and middle-income countries are subjected to physical and/or sexual intimate partner violence (IPV) in their life span. Prior studies have highlighted a range of adverse health impacts of sexual IPV. However, less is known about the link between multiple high-risk fertility behaviours and sexual intimate partner violence. The present study examines the statistical association between multiple high-risk fertility behaviours and sexual intimate partner violence among women in India.

**Methods:**

The present study used a nationally representative dataset, the National Family Health Survey (NFHS-4) 2015–16. A total of 23,597 women were included in the study; a subsample of married women of reproductive age who have had at least one child 5 years prior to the survey and who had valid information about sexual IPV. Logistic regression models were employed alongside descriptive statistics.

**Results:**

Approximately 7% of women who are or had been married face sexual IPV. The prevalence of sexual violence was higher among women who had short birth intervals and women who had given birth more than three times (12%). Around 11% of women who had experienced any high-risk fertility behaviours also experienced sexual violence. The unadjusted association suggested that multiple high-risk fertility behaviours were 32% (UORs = 1.32, 95% CI: 1.16–1.50) higher for those women who experienced sexual violence. After adjusting for other sociodemographic variables, except for women’s education and wealth quantile, the odds of multiple high-risk fertility behaviours were 16% (AOR = 1.16; 95% CI: 1.02–1.34) higher among women who faced sexual violence. The inclusion of women’s educational attainment and wealth status in the model made the association between sexual IPV and high-risk fertility behaviours insignificant.

**Conclusion:**

Sexual intimate partner violence is statistically associated with high-risk fertility behaviours among women in India. Programs and strategies designed to improve women’s reproductive health should investigate the different dimensions of sexual IPV in India.

## Background

Sexual Intimate Partner Violence (IPV) is the most common type of abuse faced by women [1]. Globally, 2018 estimates indicate that 736 million women were survivors of intimate partner violence, with 13% of women aged 15–49 years, who were or had been married or cohabitated women, having experienced physical or sexual intimate partner abuse in the last 12 months [2]. Lower- and middle-income countries see disproportionate number of women affected by violence, with 37% of women aged 15 to 49 having had exposure to physical and/or sexual intimate partner violence in their lifetime [2].

Sexual violence puts women at risk for a variety of disorders, including mental illness and post-traumatic stress disorder. These have an overall negative impact on women’s health [1, 3, 4]. Furthermore, sexual IPV increases the risk of an unplanned, mistimed, or unintended pregnancy among women, who lack access to family planning methods [5–8]. High-risk fertility behaviour is one hypothesized activity in which sexual IPV influences unwanted pregnancy and related health effects in mothers [9].

High-risk fertility behaviours have also been linked to adverse child health, like undernutrition, high child mortality or maternal mortality, and other adverse health outcomes [10–17]. Presumable determinants of high-risk fertility behaviours have been the focus of several studies. For instance, engaging in high-risk fertility behaviours is shown to be influenced by various factors, such as the mother’s level of education, rural residence, sporadic prenatal care, and contraceptive use [18, 19].

There is significant evidence suggesting that sexual assault can predict various biodemographic aspects of high-risk fertility behaviours [16, 20]. Sexual assault during adolescence can increase the likelihood of pregnancy and motherhood [21]. Studies conducted in high-income countries show that women who had experienced intimate partner abuse may be less able to space their pregnancies than women who did not [16]. According to a study, women who have been victimized by an intimate partner have a 51% chance of pregnancy and are 41% more likely to become pregnant within 18 months of the index pregnancy compared to women who did not experience any form of sexual IPV [22–24]. Personal histories of sexual and physical violence were also found to be statistically significant predictors of shorter interbirth intervals [25].

According to the National Family Health Survey (NFHS), a quarter of currently married women engages in high-risk fertility behaviours [26]. The degree to which one is likely to engage in high-risk fertility behaviours has been linked to residence, religion, education level, and marital status [15]. However, there is a scarcity of research in India focusing on the impact of sexual IPV on multiple high-risk fertility behaviours. Even though there is a link between sexual IPV and various biodemographic risk factors for high-risk fertility behaviours, to the best of our knowledge, no study has examined the link between sexual intimate partner violence and multiple high-risk fertility behaviours in India using cross-sectional nationally representative surveys. Therefore, the novelty of our paper is that we study the effect of sexual IPV on multiple high-risk fertility behaviours. The findings will provide an important foundation for future policy directions to confirm the impact of sexual abuse on multiple high-risk fertility behaviours among women in India, particularly within the marital union.

## Methods

### Data source

We used data from the 2015–16 National Family Health Survey (NFHS-4), conducted under the stewardship of the Ministry of Health and Family Welfare (MoHFW) in India. MoHFW designated the International Institute for Population Sciences (IIPS), Mumbai as the nodal agency for the survey. NFHS-4 provides essential data on health, family welfare, and other data on emerging issues in these areas like malnutrition, anaemia, hypertension, and domestic violence in India. Data is provided at the national level and is also broken down by state, union territory, and district. The NFHS-4 collects data using a stratified two-stage sampling design.

The sample for NFHS-4 included 628,900 households. Among these households, 699,628 women were selected for the interview. In India, NFHS-4 provides information at the district level, but the survey includes a separate module for ‘domestic violence’ information at the state level. The data on domestic violence was collected from only one eligible woman per household; the selected participant was randomly selected to answer the questions pertaining to the domestic violence section.

**Fig. 1:**
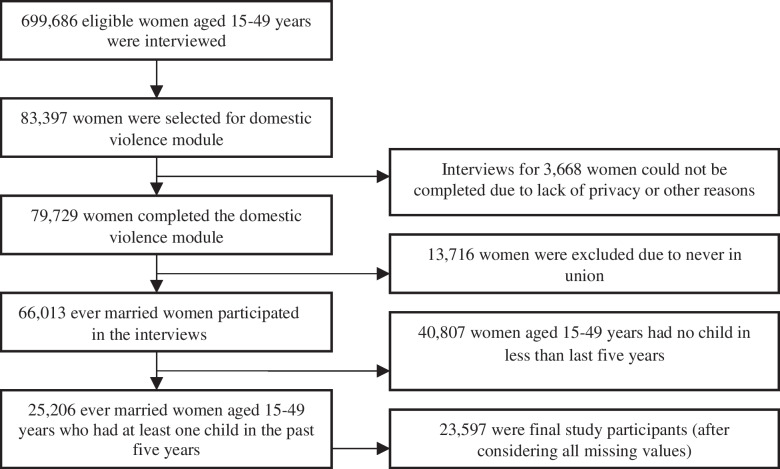
Sample size estimation for sexual violence and multiple high-risk fertility behavior in women, NFHS-4 (2015-16), India

The present study focused on ever married women aged 15–49 years. NFHS-4 selected 83,397 women for the interview in the domestic violence module. However, only 79,729 women completed the domestic violence module. In our analysis, multiple high-risk fertility behaviours were evaluated only for ever married women aged 15–49 years and who had at least one child within the five-years period preceding the survey. Therefore, the study excluded never-married women (13,716) and childless women (40,807) (Fig. 1).

### Outcome variable

In this study, women with multiple high-risk fertility behaviours were the primary outcome variable, based on the following criteria 1) women whose age at first birth was less than 18 years, 2) women who were over the age of 34 years at the time of delivery, 3) the most recent child was born within the 24 months of the prior one, and 4) latest child of order three or higher. Available yes or no responses to questions on these criteria were used in identifying the outcome variable, multiple high-risk fertility behaviours. Multiple high-risk fertility behaviours were identified in cases when responses indicated at least two of the aforementioned criteria. Multiple high-risk fertility behaviours were coded as no = 0 and yes = 1 in a binary variable.

### Independent variables

The main independent variable for this study was women who had experienced sexual IPV. The computation of sexual intimate partner violence was defined according to Demographic Health Survey criteria. For the computation of sexual IPV, three questions were asked: (1) had the respondent ever been physically forced to have unwanted sex by their husband/partner?; (2) had the respondent ever been forced into other unwanted sexual acts by their husband/partner?; and (3) had the respondent ever been physically forced to perform sexual acts they did not want to perform? The possible response options were “never,” “often,” “sometimes,” and “yes, but not in the last 12 months”. Therefore, we made a binary variable of sexual IPV where 0 was coded for responses of ‘never’ and 1 was coded for all other responses (often, sometimes, yes, but not in the last 12 months).

### Other control variables

The predictor variables were included following the literature, while control variables were considered because they had statistically significant relationships with multiple high-risk fertility behaviours [19]. These variables included the age of the women (i.e., 15–24 and 25–49 years); women’s educational attainment (i.e., no education, primary, secondary, and higher); place of residence (i.e., urban, rural); caste of the household head (i.e., Schedule Caste and Tribes, Others caste included the forward caste and the Other Backward Caste (OBC)); religious beliefs (i.e., Hindu, Muslim, and Other); wealth status (i.e, poorest, poor, middle, richer, richest quintile); women contraceptive use (i.e., no, yes); age at first sex (i.e., < 18 years, ≥ 18 years); age at first marriage (i.e., < 18 years, ≥ 18 years); possession of bank account (i.e., no, yes); and possession of a mobile phone (i.e., no, yes).

### Statistical analysis

Descriptive analyses were carried out, and the findings were presented in unweighted frequencies and weighted percentages. Then, bivariate analyses were carried out to observe the distribution of the covariates according to multiple high-risk fertility behaviours. Pearson’s chi-square statistics was also applied to test the hypothesis. In order to determine the association of sexual intimate partner violence and multiple high-risk fertility behaviours, unadjusted and adjusted multivariable logistic regressions were conducted. The significant level of the variables is < 0.05. Both unadjusted odds ratios (UORs) and adjusted odds ratios (AORs) were reported with 95% confidence intervals (CIs). We also examined the association between multiple high-risk fertility behaviours and sexual IPV to control the other predictor variables through the different models. Model 1 reports the unadjusted sexual IPV odds ratios. Model 2 adjusts only sexual IPV, women’s education, and wealth variables. Model 3 shows the odds of sexual IPV, adjusted by age group, women’s working status, bank account, mobile phone, contraception method, residence, caste, religion, and region. Model 4 adjusts with other confounders. We have used the appropriate sampling weight for the domestic violence module [27]. All the analyses were done using STATA-14.0 (College Station, Texas, USA).

## Results

Table 1 shows the demographic and socioeconomic characteristics of the sample as well as the prevalence of sexual intimate partner violence. Most participants were aged 25–49 years. Around half of the women in the sample had received secondary education. More than a third of respondents were from rural areas. Respondents from other castes constituted around 69% of the sample. Most of the women in the sample believed in the Hindu religion, whereas 14% believed in Islam. Around 23% of the sample households belonged to the poorer wealth quantile. Around half of the women did not use contraception. Approximately 35% of the women had sex before turning 18 years old.

**Table 1:** Distribution of respondents by background characteristics and prevalence of Sexual Intimate Partner Violence (Sexual IPV) by socioeconomic characteristics in India

Variables	n (%)	Sexual IPV		***p***-value
		No	Yes	
**Age group**				
15–24	6660 (34.2)	93.1	6.9	0.722
25–49	16,937 (65.8)	92.6	7.4	
**Women education**				
No education	6733 (26.7)	88.9	11.1	< 0.000
Primary	3279 (12.8)	90.2	9.8	
Secondary	10,964 (47.5)	94.3	5.7	
Higher	2621 (13.1)	97.4	2.6	
**Residence**				
Urban	6240 (30.6)	95.4	4.6	< 0.000
Rural	17,357 (69.4)	91.6	8.4	
**Caste**				
Schedule caste & Tribes	9581 (31.3)	90.5	9.5	< 0.000
Others caste	14,016 (68.6)	93.8	6.2	
**Religion**				
Hindu	17,585 (80.9)	92.8	7.2	< 0.006
Muslim	3024 (14.1)	92.3	7.7	
Others	2988 (5.0)	94.2	5.8	
**Wealth**				
Poorer	5842 (22.7)	87.1	13.0	< 0.000
Poor	5271 (20.4)	92.1	7.9	
Middle	4769 (20.2)	93.4	6.6	
Richer	4100 (18.8)	94.9	5.1	
Richest	3615 (17.4)	97.8	2.2	
**Contraception usage**				
Not using	13,150 (55.5)	92.7	7.2	< 0.295
Using	10,447 (44.5)	92.7	7.2	
**Age at first sex**				
< 18 years	7517 (35.0)	90.5	9.5	< 0.000
≥18 years	15,259 (65.0)	94.1	5.9	
**Age at first marriage**				
< 18 years	7873 (36.2)	90.5	9.4	< 0.000
≥18 years	15,434 (63.9)	94.1	5.8	
**Bank account**				
No	11,849 (50.4)	91.5	8.4	< 0.000
Yes	11,748 (49.6)	93.9	6.0	
**Mobile phone**				
No	11,778 (49.5)	91.4	8.5	< 0.000
Yes	11,819 (50.5)	94.0	5.9	
**Total**	23,597	21,961 (92.7)	1636 (6.9)	

Further in Table 1, the prevalence of sexual intimate partner violence by background characteristics is presented. Sexual IPV is reported among 11% of women without any formal education; however, only 3% of women with higher education reported experiencing sexual IPV. The prevalence of sexual IPV is more common among women living in rural areas. When it comes to caste, the Scheduled Caste and Tribes women have a sexual IPV rate of 10%. In other religious groups, the prevalence of sexual intimate partner violence was substantially lower, roughly at 6%. Sexual IPV is prevalent among roughly 13% of poorer women and 2% of richer women. Sexual IPV was reported by 10% of women under the age of 18 who have had sex, compared with only 6% who had sex for the first time when over the age of 18. Among women without a bank account, 9% experienced sexual intimate partner violence. Sexual IPV was reported by 6% of women who were mobile phone owners.

The prevalence of multiple high-risk fertility behaviours can also be seen in Table 2. Multiple high-risk fertility behaviours affect around 13% of women. Prevalence of multiple sexual behaviours is 17% among women who experienced sexual intimate partner violence. Multiple high-risk fertility behaviours are more common in cases of women aged 25–49, with 17% reporting it. Multiple high-risk fertility behaviours are also more common in the group of women with no education, while only 4% of higher educated women engaged in multiple high-risk fertility behaviours. Multiple high-risk sexual behaviours were significantly more common in rural women (around 14%) than in urban women (approximately 9%). Among women from scheduled tribes (STs) 16% indicated multiple high-risk fertility behaviours, higher than other caste group. About 20% of Muslim women experienced multiple high-risk fertility behaviours, while women from other religions experienced less multiple high-risk fertility behaviours. High-risk fertility affects 24% of the poorest women. Women who used contraception had a 6% prevalence of multiple high-risk fertility behaviours. Around 14% of respondents who do not have a bank account have high risks of fertility behaviours. The prevalence of high-risk fertility behaviours among women who own a mobile phone was 9%.

**Table 2:** Prevalence of multiple high-risk fertility behaviours by socioeconomic characteristics

Variables	Any multiple high-risk fertility behaviours		***p***-value
	No	Yes	
**Sexual IPV**			
No	87.9	12.2	< 0.000
Yes	82.8	17.2	
**Age group**			
15–24	96.5	3.6	< 0.000
25–49	82.8	17.2	
**Women education**			
No education	73.9	26.1	< 0.000
Primary	86.5	13.5	
Secondary	93.0	7.0	
Higher	96.1	3.9	
**Residence**			
Urban	91.4	8.6	< 0.000
Rural	85.8	14.2	
**Caste**			
Schedule caste & Tribes	85.3	14.7	< 0.000
Others caste	88.5	11.5	
**Religion**			
Hindu	88.6	11.4	< 0.000
Muslim	79.9	20.1	
Others	90.1	9.9	
**Wealth**			
Poorer	76.2	23.8	< 0.000
Poor	85.5	14.5	
Middle	90.8	9.2	
Richer	93.4	6.6	
Richest	94.1	5.9	
**Contraception usage**			
Not using	82.6	17.4	< 0.000
Using	93.6	6.4	
**Age at first sex**			
< 18 years	83.9	16.1	< 0.000
≥18 years	89.5	10.5	
**Age at first marriage**			
< 18 years	84.7	15.3	< 0.000
≥ 18 years	89	11.0	
**Bank account**			
No	86.2	13.8	< 0.000
Yes	88.8	11.2	
**Mobile phone**			
No	83.9	16.1	< 0.000
Yes	91.0	9.0	
**Total**	19,893 (87.5)	3704 (12.5)	

**Fig. 2:**
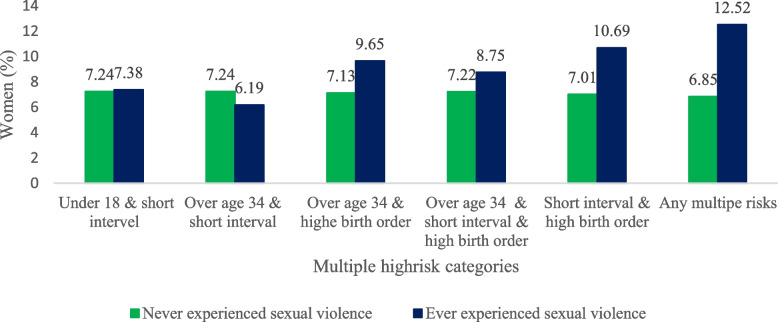
Prevalence of sexual violence by women’s multiple high-risk fertility behaviours in India

The prevalence of sexual IPV by women's multiple high-risk fertility behaviors among ever married women is depicted in Fig. 2. Sexual IPV was found in 11% of women with short interval and higher order births. Around 13% of women have experienced sexual IPV in any multiple high-risk categories.

Table 3 shows the odds of multiple high-risk fertility behaviours that emerged following the two-step regression model approach. Model 1 shows that women who have experienced sexual IPV are 32% (OR:1.32; 95% CI: 1.16–1.50) more likely to engage in multiple high-risk fertility behaviours than women who have not experienced sexual IPV. Model 2 depicts that respondent’s age, women’s education, residence, caste, religion, and wealth are significant predictors of multiple high-risk fertility behaviours, but sexual IPV is not. There is a strong endogeneity of education and wealth with sexual IPV in model 3. In the case of Model 4, the study combined Model 3 variables, except for women’s education and wealth, with other variables to find significant predictors of multiple high-risk fertility behaviours among survivors of sexual intimate partner violence. The adjusted OR of the sexual IPV shows that women who faced sexual IPV have 19% (OR:1.19; 95% CI:1.03–1.37) more likelihood to experience multiple high-risk fertility behaviours. Women in the older reproductive age group are 7.37 times (OR:7.37;95%CI:6.47–8.39) more likely to experience multiple high-risk fertility behaviours, in comparison to the reference 15–24 age category. The women residing in rural areas were 37% (OR:1.37;95%CI:1.24–1.50) more likely to engage in multiple high-risk fertility behaviours than urban women. Muslim women were 2.27 times (OR:2.27;95% CI:2.04–2.53) more likely, and other religion women were 1.68 times (OR:1.68;95% CI:1.49–1.88) more likely to engage in multiple high-risk fertility behaviours than Hindu women. The women who use contraception are 70% (OR:0.30;95% CI:0.27–0.33) less likely to engage in multiple high-risk fertility behaviours. The age at first sex and women who have mobile phone are also significant predictors for multiple high-risk fertility behaviours.

**Table 3:** Logistic regression estimate of multiple high-risk fertility behaviours by socioeconomic characteristics in India, 2015–16

Variables	Model 1	Model 2	Model 3	Model 4
	OR (95% CI)	OR (95% CI)	OR(95%CI)	OR (95% CI)
**Sexual IPV**				
No	Ref.	Ref.	Ref.	Ref.
Yes	1.32***(1.16–1.50)	1.05 (0.91–1.19)	1.06 (0.92–1.23)	1.19*(1.03–1.37)
**Age group**				
15–24		Ref.	Ref.	Ref.
25–49		5.05***(4.46–5.71)	6.34***(5.56–7.22)	7.37***(6.47–8.39)
**Women Education**				
No education		Ref.	Ref.	–
Primary		0.65***(0.58–0.72)	0.67***(0.59–0.75)	–
Secondary		0.41***(0.38–0.46)	0.43*** (0.38–0.48)	–
Higher		0.27***(0.22–0.33)	0.26***(0.21–0.32)	–
**Residence**				
Urban		Ref.	Ref.	Ref.
Rural		0.94*(0.85–1.05)	0.95 (0.85–1.06)	1.37***(1.24–1.50)
**Caste**				
Schedule caste & Tribes		Ref.	Ref.	Ref.
Others caste		0.88***(0.81–0.95)	0.90*(0.82–0.98)	0.75***(0.69–0.82)
**Religion**				
Hindu		Ref.	Ref.	Ref.
Muslim		2.01***(1.81–2.23)	1.93***(1.73–2.16)	2.27***(2.04–2.53)
Others		2.19***(1.96–2.44)	2.03***(1.80–2.28)	1.68***(1.49–1.88)
**Wealth**				
Poorer		Ref.	Ref.	Ref.
Poor		0.83***(0.76–0.92)	0.90*(0.81–1.00)	–
Middle		0.59***(0.52–0.66)	0.65***(0.58–0.74)	–
Richer		0.48***(0.42–0.56)	0.57***(0.49–0.67)	–
Richest		0.47***(0.39–0.56)	0.60***(0.49–0.72)	–
**Contraception usage**				
Not using			Ref.	Ref.
Using			0.31***(0.29–0.34)	0.30***(0.27–0.33)
**Age at first sex**				
< 18 years			Ref.	Ref.
≥ 18 years			0.81 (0.69–0.94)	0.65***(0.56–0.76)
**Age at first marriage**				
< 18 years			Ref.	Ref.
≥ 18 years			1.06***(0.91–1.23)	0.99 (0.85–1.15)
**Bank Account**				
No			Ref.	Ref.
Yes			1.16***(1.07–1.26)	0.99 (0.91–1.07)
**Mobile Phone**				
No			Ref.	Ref.
Yes			0.82***(0.75–0.90)	0.58***(0.53–0.63)
R2	0.0008	0.13	0.17	0.14

a) Ref=reference category. b) significant levels *p<0.05* ; **p<0.01; ***p<0.001

## Discussion

In contrast to the commonly used approach which tries to investigate the association between sexual IPV and one or two specific aspects of women’s reproductive health and fertility behaviours [1, 23, 24, 28], we applied a new framework to reveal the social and physical impact of domestic violence. The novelty of our paper is the use of the category of high-risk fertility behaviour, which we believe captures the complexity of fertility-related effects better than separate analyses.

Using a cross-sectional dataset, this study investigated the prevalence of multiple high-risk fertility behaviours in relation to sexual intimate partner violence in India. Our results suggest that women who have experienced sexual IPV are more likely to be exposed to multiple high-risk fertility behaviours. The latter includes women, who gave birth at ages less than 18 or above 34, whose birth interval was less than 24 months, or those who had a high birth order. Our results on the relationship between sexual intimate partner violence and high-risk fertility behaviours resonate with those research results, which revealed some specific aspects (unintended pregnancy, limited access to contraception, terminated pregnancy, infant mortality) of fertility-related impacts [21, 22, 25, 29, 30].

It is also worth noting that changing women’s education and wealth quintile can directly diminish the link between sexual abuse and high-risk fertility behaviours, according to our findings. The education level and wealth quintile of respondents are negatively associated with the high-risk fertility behaviours, and additionally they diminish the association between sexual IPV and high-risk fertility behaviours. This is possibly because illiteracy, in addition to low socioeconomic status, may put women at risk of sexual violence [31]. Thus, improving women’s status through education and increasing household wealth not only reduces physical and sexual violence against women but also has a trickle-down effect on high-risk fertility behaviours [32].

We obtained that women in the 25–49 age group are more likely to experience multiple high-risk fertility behaviours. According to one study, this could be because women in later stages of their reproductive lives are less likely to seek maternal healthcare services, and because of the lack of reproductive health knowledge that increases the likelihood of pregnancy and childbirth in women aged 35 and above [33].

Our findings on sexual intimate partner violence and short delivery intervals are similar to findings of previous studies [25, 34, 35]. Research based on Demographic and Health Survey (DHS) data found a substantial link between sexual intimate partner violence and unplanned pregnancies and live births within 24 months of the previous one [34]. In addition, using the DHS dataset, [25] observed that individual history of sexual IPV and village-level prevalence of sexual IPV were linked to shorter intervals between live births in multilevel research. Forced sexual intercourse is one possible explanation for the shorter intervals between live births among women subjected to sexual violence. According to studies, women who are subjected to sexual violence engage in unprotected sexual activity, such as not using a condom during sex, which can result in unwanted pregnancy and shorter intervals between live births [36]. On the other hand, there are evidences, which emphasize the complexity of the relationship between sexual IPV and fertility and it has been found that infertility itself increases the risk of domestic violence [37, 38]. However, after controlling for several variables, our findings indicate that sexual IPV is strongly associated with high-risk fertility behaviours.

Furthermore, our research showed that women, who used contraceptives had a lower likelihood of engaging in multiple high-risk fertility behaviours compared to women, who did not use contraceptives. Planned birth intervals and the suppression of unwanted and mistimed births are two of the fundamental goals of contraceptive use [13, 39]. Moreover, women who use the mobile phone was also found to have lower high-risk fertility behaviours. According to studies, women’s autonomy, in many forms, can directly influence women’s decision-making and fertility behaviours [17–19].

To the best of our knowledge, no previous research has investigated the impact of sexual intimate partner violence on high-risk fertility behaviours. Findings of the present study could contribute to designing intervention policies for women in abusive marriages in India, reducing the negative impact of sexual violence on high-risk fertility behaviours. The emphasis on the relationship between sexual IPV and fertility behaviours has strong policy implications, too. Since the reproductive health care unit is the only contact to the health care system for several women in India, these clinics should be involved in unveiling and reporting sexual intimate partner violence [30]. Providing counselling programs on partner violence by reproductive healthcare units could contribute to reducing domestic violence and protecting the potential victims [40]. In addition to the involvement of the reproductive health units in the reporting process, the mandatory reporting of domestic violence could improve the unveiling of sexual violence [41, 42].

The current study and its conclusions, however, should be viewed with a few caveats in mind. It should be considered in the interpretation, that a few questions can not measure all the dimensions of sexual IPV. No causal implications could be drawn, because the current study is based on a cross-sectional dataset. The sexual IPV module of the survey was only given to randomly selected ever-married women aged 15 to 49 in the National Family Health Survey [26]. As a result, our findings do not apply to women who have never married. However, a partial mediation effect based on women’s autonomy can be used to analyse the link between sexual violence and several high-risk reproductive behaviours. Future prospective studies are needed to investigate the role of women’s autonomy in mediating the relationship between sexual IPV and several high-risk fertility behaviours, as well as possible mediators in this relationship.

## Conclusion

Our findings indicate that sexual intimate partner violence is associated with high-risk fertility behavior among Indian women. Sexual IPV and its various manifestations in India should be considered when designing and implementing policies aimed at improving women’s reproductive health. It is well established that women’s autonomy and empowerment can redefine sexual rights and increase women awareness of high-risk fertility behaviours, which suggests that improvement of women’s socioeconomic status through education and employment opportunities should be key policy objectives. There is a need for increased awareness of sexual IPV and high fertility behaviours, and women’s empowerment via education, contraceptive usage, and employment may contribute to establishing these circumstances.

## Data Availability

The present study used the dataset available in the public domain. For more details, visit at www.measuredhs.com and can be accessed after the request is made to and approved by the DHS program.
