# Correction: Does sexual Intimate Partner Violence (IPV) increase risk of multiple high-risk fertility behaviours in India: evidence from National Family Health Survey 2015–16

**DOI:** 10.1186/s12889-022-14742-0

**Published:** 2022-12-20

**Authors:** Milan Das, Csaba G. Tóth, Neha Shri, Mayank Singh, Babul Hossain

**Affiliations:** 1 International Institute for Population Sciences, Mumbai, India; 2 Research Fellow, Centre for Economic and Regional Studies, Budapest, Hungary; Corvinus Institute for Advanced Studies, Budapest, Hungary

**Correction: BMC Public Health 22, 2081 (2022)**

**https://doi.org/10.1186/s12889-022-14289-0**

The original publication of this article [1] contained an error in the P-values in table 1 and 2, “<0.000” should be “<0.001”. This does not affect the conclusions/results of the article. The original article has been updated.
